# Association between hyperuricemia and diabetic nephropathy: insights from the national health and nutrition examination survey 2007–2016 and mendelian randomization analysis

**DOI:** 10.1007/s11255-024-04094-6

**Published:** 2024-05-29

**Authors:** Sensen Wu, Hui Wang, Dikang Pan, Julong Guo, Fan Zhang, Yachan Ning, Yongquan Gu, Lianrui Guo

**Affiliations:** 1 Department of Vascular Surgery, Xuanwu Hospital, Capital Medical University, Beijing, China; 2 Department of Intensive Care Medicine, Xuanwu Hospital, Capital Medical University, Beijing, China

**Keywords:** Uric acid, Diabetic nephropathy, National Health and Nutrition Examination Survey, Mendelian Randomization

## Abstract

**Background:**

This study aimed to investigate the role of uric acid (UA) in diabetic nephropathy (DN) from epidemiological and genetic perspectives.

**Methods:**

We used data from the 2007–2016 National Health and Nutrition Examination Survey to evaluate the relationship between UA and DN risk using weighted multivariate-adjusted logistic regression. Subsequently, a two-sample Mendelian randomization study was conducted using genome-wide association study summary statistics. The main inverse variance weighting (IVW) method and supplementary MR method were used to verify the causal relationship between UA and DN, and sensitivity analysis was conducted to confirm the credibility of the results.

**Results:**

Our observational study enrolled 4363 participants with diabetes mellitus from NHANES, among them, 2682 (61.4%) participants were identified as DN. The multivariate logistic regression model showed that compared with those without hyperuricemia, the DN risk of the hyperuricemia population was significantly increased (*P* < 0.05). The MR results suggest a direct causal effect of hyperuricemia on DN (IVW odds ratio (OR): 1.37 (95% confidence interval 1.07–1.76); *P* = 0.01), which is consistent with findings from other MR methods.

**Conclusion:**

The evidence from observational studies indicates a positive correlation between HUA and the onset of DN. And the causal effects of HUA on DN were supported by the MR analysis.

**Supplementary Information:**

The online version contains supplementary material available at 10.1007/s11255-024-04094-6.

## Introduction

According to the ninth edition of data from the International Diabetes Federation (IDF), the global prevalence of diabetes in 2019 was 9.3%, affecting approximately 463 million people. This is expected to rise to 10.2% (578 million people) by 2030, and further increase to 10.9% (700 million people) by 2045. The rise in diabetes patients and the subsequent occurrence of related complications has created a significant demand for social, hospital, and financial resources [1].

Diabetic nephropathy (DN) is a common complication of diabetes and a leading cause of end-stage renal disease. It is estimated that nearly 40% of diabetes patients worldwide will develop diabetic nephropathy [2]. DN is characterized by pathological excretion of albumin in urine, glomerulopathy and reduction of glomerular filtration rate (GFR) [3]. Several studies [4, 5] have reported that diabetes nephropathy is related to the onset of cardiovascular disease, and cardiovascular disease will worsen the overall prognosis.

The pathogenesis of diabetic nephropathy is complex and has not yet been fully elucidated. Existing studies have identified several risk factors in the development of diabetic nephropathy, including endothelial dysfunction, smoking, stimulation of the renin-angiotensin system, obesity, and hypertension [6, 7]. Additionally, it has been reported that serum uric acid (SUA) is associated with kidney disease, and there is a belief that hyperuricemia (HUA) may serve as a marker for diabetes microvascular disease or be a causative factor in diabetes arteriolar disease itself [8]. However, some studies have found no significant relationship between SUA and DN [9, 10]. Therefore, there is still disagreement on whether hyperuricemia is solely a marker of DN or a contributing factor to diabetes microvascular disease and nephropathy.

Existing observational studies have limitations, such as small sample sizes and insufficient adjustment for important covariates. The National Health and Nutrition Examination Survey (NHANES) is an ongoing cross-sectional study conducted by the National Center for Health Statistics (NCHS) in the United States, aimed at assessing the nutritional status and emerging public health of the US population [11]. Therefore, NHANES can provide high-quality, large-sample, nationally representative data to assess the correlation between hyperuricemia and the risk of diabetic nephropathy.

Mendelian randomization (MR) analysis is a method that uses instrumental variables, such as single nucleotide polymorphisms (SNPs), to elucidate the causal relationship between exposure and outcomes [12]. Compared with traditional observational studies, MR analysis has the advantages of avoiding environmental confounding factors and eliminating reverse causal relationships, and it can effectively reduce bias, similar to randomized controlled trials (RCT) research, using statistical methods that incorporate causal relationships [13].

In this study, we hypothesized that HUA may affect renal function in patients with diabetes. Initially, we conducted a cross-sectional survey using a comprehensive NHANES database. Subsequently, we plan to use MR methods to delve into the field of genetics and evaluate the causal relationship between HUA and DN. This stage aimed to reveal the correlation between HUA and DN risk, providing information for clinical strategies for DN prevention.

## Materials and methods

### Cross-sectional study design

#### Database and study subjects

NHANES is a nationally representative survey that assesses the health and nutrition of the non-hospitalized population in the United States. The survey includes interviews, physical examinations, and laboratory tests, and has been releasing data every two years since 1999. NHANES is designed and managed by the National Center for Health Statistics and is approved by its Research Ethics Review Committee. Can visit (http://www.cdc.gov/nchs/NHANES.htm.) for more information about the NHANES database.

In this study, we analyzed NHANES data for adults aged 18 and older from 2007 to 2016. We collected information on participants’ age, gender, race, education level, poverty-to-income ratio (PIR), marital status, smoking status, body mass index (BMI), and chronic diseases. Additionally, laboratory indicators such as urinary protein, urinary creatinine, glycated hemoglobin, and SUA were collected from participants. Participants who lacked the aforementioned information were excluded.

#### Assessment of diabetes and diabetic nephropathy

In our study, diabetes is defined as (1) a diagnosis by doctors; (2) the use of hypoglycemic drugs or insulin; and (3) fasting blood glucose levels higher than 7.0 mmol/L (126 mg/dL) or glycated hemoglobin A1c (HbA1c) levels higher than 6.5% [14]. The diagnosis of diabetic nephropathy is determined by the presence of urinary albumin-to-creatinine ratio (UACR) ≥ 300 mg/g or estimated glomerular filtration rate (eGFR) < 60 ml/min/1.73m [15]. Hyperuricemia is diagnosed in men with serum uric acid levels greater than 420 μmol/L and in women with levels greater than 360 μmol/L.

#### Other covariates

Other demographic characteristics in NHANES are determined through self-reported data. These include age (in years) and gender (male or female). Additionally, race is classified as Mexican American, non-Hispanic white, non-Hispanic black, other Hispanic, or other races. Education level is categorized as less than high school, high school or equivalent, and university or higher education in our study. Marital status (grouped as married or living with a partner, married, divorced or separated, and unmarried), and smoking status were also considered. Non-smokers are defined as participants who have smoked fewer than 100 cigarettes in their lifetime, while current smokers are individuals who have smoked more than 100 cigarettes but have not quit. Former smokers are those who had previously smoked more than 100 cigarettes but have since quit.

### MR study design

#### Study design and data source

The MR method relies on three basic assumptions for causal inference: (1) There must be a correlation between instrumental variables (IVs, commonly genetic variations) and exposure factors. (2) IVs should only affect the observed results through exposure factors and should not be associated with other confounding factors. (3) The allocation of IVs should be random and not influenced by the observed results. The following steps will help in selecting the best IVs related to DN.

Given that population mixing may lead to biased estimates, we limited the genetic background of the population in the MR study to individuals of European ancestry, comprising 312,650 European individuals (4,111 cases and 308,539 controls).

#### Selection of genetic instruments

In order to test the initial hypothesis, this study collected Genome-wide significant (*P* < 5 × 10^–8^) SNPs that predict SUA in a population of European ancestry. To ensure the independence of the instruments, a linkage disequilibrium (LD) clumping algorithm was applied, using a stringent cut-off value of r^2^ < 0.01 [16]. Each SNP’s F statistic (F = beta2/se2) was utilized to evaluate the power of the remaining SNPs, and those with an F value < 10 were excluded from subsequent analysis. Harmonization was employed to exclude palindromic and incompatible SNPs. The MR-Pleiotropy Residual and Outlier (MR-PRESSO) test was utilized to detect and exclude any outlier SNPs.

### Statistical analysis

For NHANES cross-sectional studies, continuous variables are reported as mean ± standard deviation, while categorical variables are presented as case (n) and percentage (%). Differences were tested using the chi-square test for categorical variables, the one-way ANOVA test for normal distribution, or the Kruskal–Wallis test for skewed distribution. Logistic regression was used to calculate the odds ratio (OR) and 95% confidence interval (CI) of the correlation between HUA and DN.

In the MR analysis, the primary method used was the Inverse-Variance Weighted (IVW) method. Multiple complementary MR detection methods were employed to ensure robustness and accuracy in examining causal effects and correcting for the potential impact of horizontal pleiotropy. These methods included the Weighted Median Method, Weighted Mode Method, Simple Mode, MR-Egger Regression Method, and MR-PRESSO. These diverse approaches collectively enhanced the reliability of the causal inference drawn from the analysis [17]. Besides, Cochrane’s Q test was used to assess the heterogeneity among estimates of SNPs in one analysis. This MR study was reported according to the STROBE-MR checklist [18].

All analyses were conducted using Stata 17.0 (Stata Corporation, College Station, TX, USA) and R (version 4.3.1). A significance threshold of *p* < 0.05 was employed to determine statistical significance in all analyses.

## Results

**Fig. 1:**
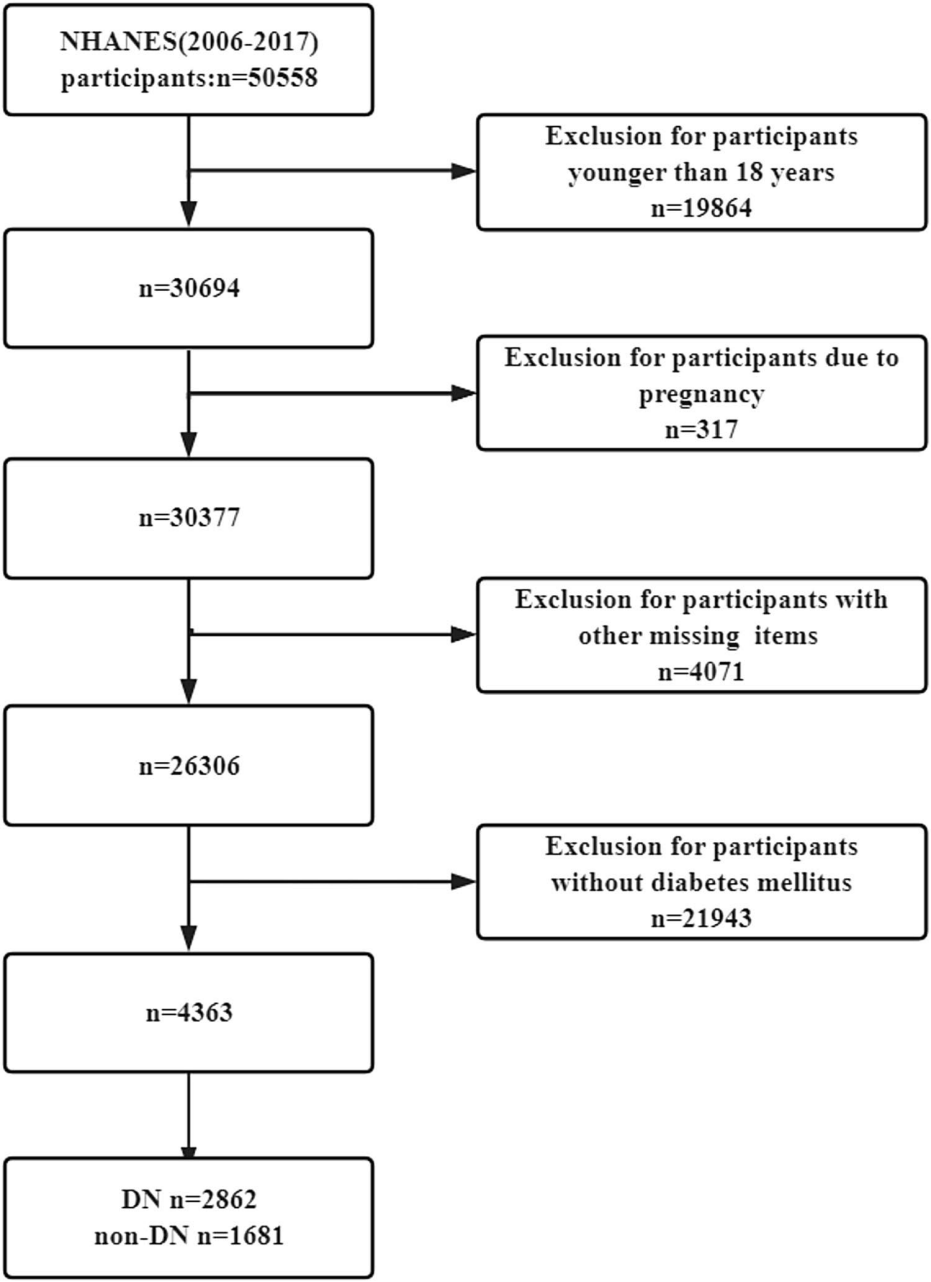
The flow diagram of participant selection

From 2007 to 2016, a total of 50,588 individuals participated in NHANES. After exclusions, 4363 participants with diabetes mellitus (DM) were included in the final analysis (Fig. 1), and their main characteristics are detailed in Table 1. Among them, 2682 (61.4%) participants were identified as having renal insufficiency. Table 1 provides important insights into the demographic and clinical characteristics of the study population. The distribution of sex was found to be significantly different between the two groups (*p* = 0.04), with a relatively high proportion of males in both groups. Age and race also demonstrated significant differences between the groups (*p* < 0.001 for both). Additionally, smoking status, presence of hypertension, hypercholesterolemia, coronary heart disease, stroke, and several laboratory parameters including urine protein, blood urea nitrogen (BUN), and SUA levels were found to be significantly different between the groups (all *p* < 0.001). As shown in the table, the proportion of hyperuricemia in patients with diabetic nephropathy is significantly higher (35.87% vs 14.40%, *p* < 0.001), with average SUA levels of 364 ± 98 vs 313 ± 78 umol/L (*p* < 0.001).

**Table 1:** Patient demographics and baseline characteristics

Characteristic	Diabetic nephropathy		*p*-value
	No *N* = 1681	Yes *N* = 2682	
Male, *n*(%)	923 (54.91%)	1387 (51.72%)	0.040
Age, years	54 ± 13	65 ± 12	< 0.001
BMI (kg/m^2^)	32 ± 7	32 ± 7	0.292
PIR	2.30 ± 1.50	2.22 ± 1.43	0.066
Drink, *n* (%)	895 (53.24%)	1,184 (44.15%)	< 0.001
*Smoke status*, *n* (%)			< 0.001
Current smoker	342 (20.35%)	398 (14.84%)	
Former smoker	479 (28.49%)	971 (36.20%)	
Never	860 (51.16%)	1,313 (48.96%)	
Hypertension, *n* (%)	894 (53.18%)	1,961 (73.12%)	< 0.001
Hypercholesterolemia, *n* (%)	923 (54.91%)	1,675 (62.45%)	< 0.001
CAD, *n* (%)	156 (9.28%)	575 (21.44%)	< 0.001
Stroke, *n* (%)	62 (3.69%)	290 (10.81%)	< 0.001
Urinary protein, mg/L	12 ± 11	231 ± 917	< 0.001
UCr, mg/dL	114 ± 70	118 ± 74	0.078
HGB g/dL	14.15 ± 1.52	13.58 ± 1.64	< 0.001
HbA1c %	7.35 ± 1.76	7.39 ± 1.76	0.444
TC, mmol/L	4.91 ± 1.11	4.75 ± 1.21	< 0.001
TG, mmol/L	2.22 ± 1.99	2.18 ± 1.73	0.504
HDL, mmol/L	1.23 ± 0.37	1.24 ± 0.38	0.221
Albumin, g/L	42.0 ± 3.3	41.2 ± 3.5	< 0.001
ALT, U/L	30 ± 23	26 ± 35	< 0.001
AST, U/L	28 ± 16	27 ± 25	0.425
TBIL, umol/L	11.4 ± 5.6	11.3 ± 4.8	0.488
BUN, mmol/L	4.48 ± 1.38	6.54 ± 3.35	< 0.001
UA, umolL	313 ± 78	364 ± 98	< 0.001
HUA	242 (14.40%)	962 (35.87%)	< 0.001

Number and proportion were presented for categorical variables, mean and standard deviation were presented for continuous variables

*BMI*   Body Mass Index, *PIR* Poverty Income Ratio, *CAD*  Coronary Artery Disease, *HGB*   Hemoglobin, *HbA1c*   Glycated Hemoglobin, *TC*   Total Cholesterol, *TG*   Triglyceride, *HDL*   High-density lipoprotein, *ALT*   Alanine aminotransferase, *AST * Aspartate aminotransferase, *TBIL*  Total bilirubin; BUN = Blood Urea Nitrogen, *UA*   Uric Acid, *HUA*   hyperuricemia, *UCr*   Urine Creatinine

Table 2 presents the results of weighted multivariate-adjusted logistic regression in four models. In the unadjusted Model 1, hyperuricemia showed significant associations with DN (OR 3.33, 95%CI 2.84–3.89, *P* < 0.001). After adjusting for covariates, there was no significant change in this association. In Model 2, Model 3, and Model 4, it is evident that hyperuricemia is significantly correlated with DN, with OR values of 2.79 (95% CI 2.34–3.32), 2.78 (95% CI 2.36–3.27), and 2.56 (95% CI 2.11–3.12), all *P* < 0.001, respectively.

**Table 2:** Weighted multivariable-adjusted logistic regression of association between HUA and incident DN

Variables	Model1		Model2		Model3		Model4	
	OR (95%CI)	*P*	OR (95%CI)	*P*	OR (95%CI)	*P*	OR (95%CI)	*P*
*HUA*								
No	1.00 (Reference)		1.00 (Reference)		1.00 (Reference)		1.00 (Reference)	
Yes	3.33 (2.84–3.89)	** < .001**	2.79 (2.34–3.32)	** < .001**	2.78 (2.36–3.27)	** < .001**	2.56 (2.11–3.12)	** < .001**

*OR* Odds Ratio, *CI* Confidence Interval, *BMI*  Body Mass Index, *PIR*  Poverty Income Ratio, *CAD*  Coronary Artery Disease, *HGB*  Hemoglobin, *HbA1c* Glycated Hemoglobin; *TC* Total Cholesterol; *TG* Triglyceride; *HDL* High-density lipoprotein; *ALT* Alanine aminotransferase; *AST* Aspartate aminotransferase; *TBIL* Total bilirubin, *BUN* Blood Urea Nitrogen; *UA* Uric Acid; *HUA* hyperuricemia, *UCr*  Urine Creatinine

Model 1: crude

Model 2: adjusted for PIR, BMI, age, sex, race, education and marriage

Model 3: Model 2 + adjusted for drink, smoke, hypertension, hypercholesterolemia, CAD, stroke

Model 4: Model 3 + adjusted for laboratory tests include urine protein, urine creatinine, HgB g/dL, glycated hemoglobin, TC, TG, HDL, TBIL, albumin, ALT, AST, BUN, etc

**Fig. 2:**
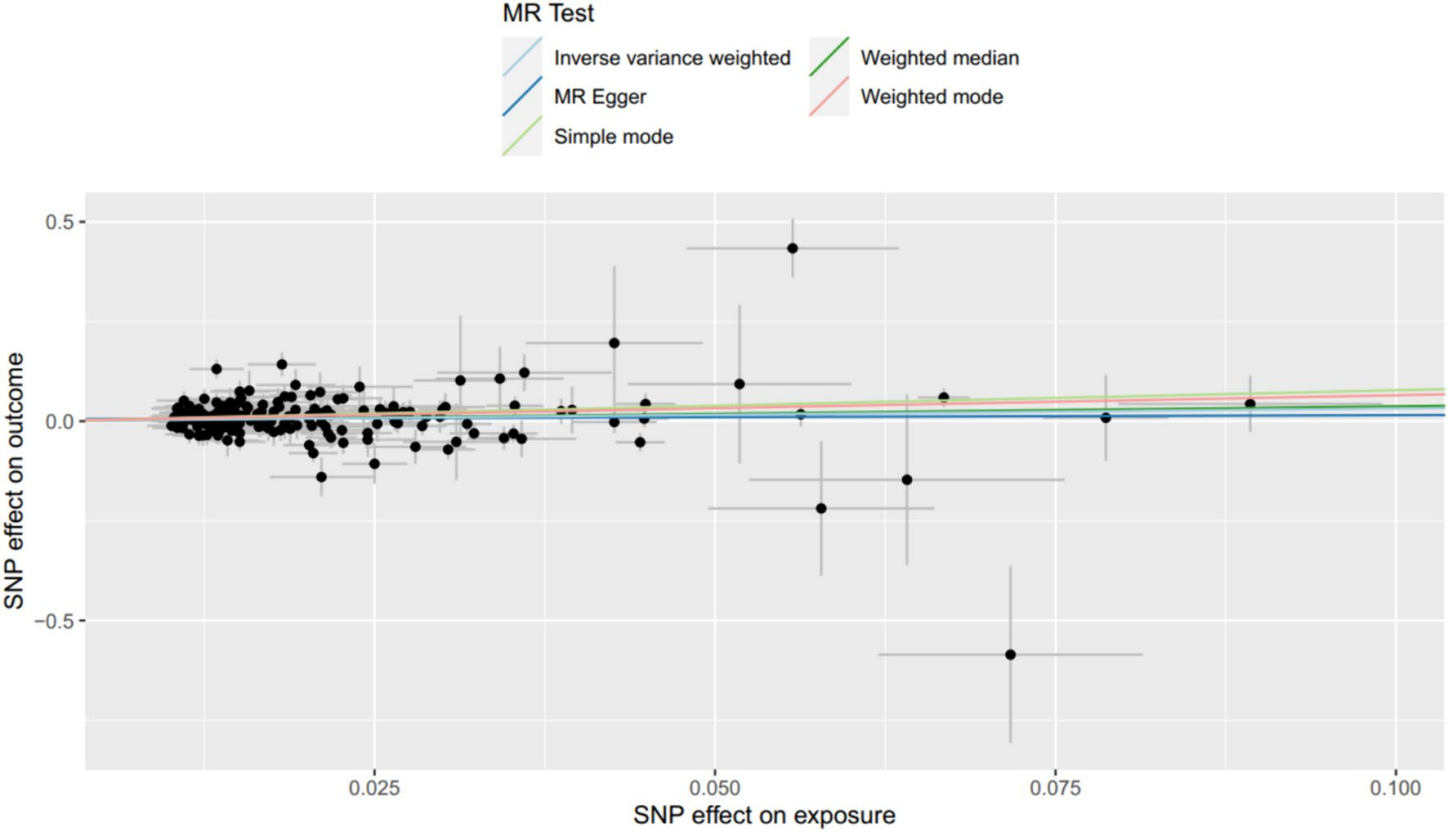
Scatter plots of primary MR analysis. The slope of each line corresponds to the estimated MR effect in different models

Based on the predetermined criteria, 223 SNPs significantly related to diabetic nephropathy and blood uric acid were included for further analysis (Table S1), with 190 SNPs included after the deletion of some missing values. Please refer to Table 3 for the analysis results. Notably, all F statistics exceeded 10, indicating a relatively low risk of weak instrument bias in the conducted MR analyses. The results of the univariable MR analysis, which aimed to explore the causal effect of HUA on DN, are presented in Fig. 2. The scatter plots and forest plots are depicted in Figure S1. Significant heterogeneity was observed, as indicated by Cochran’s Q test (*p* < 0.001), and the main analyses were conducted using the IVW approach with the multiplicative random-effect model. The results suggest a direct causal effect of HUA on DN (IVW odds ratio (OR): 1.37 (95% confidence interval 1.07–1.76); *P* = 0.01), which is consistent with findings from other MR methods. Subsequently, the MR-PRESSO test was conducted, and the outlier-corrected result (*P* = 0.018) after removing outlier single nucleotide polymorphisms (SNPs) aligned with the IVW outcome. The MR-Egger regression intercept term indicated no apparent directional pleiotropy among the SNPs in the datasets (*P* = 0.72), and the symmetry of the funnel plot supported the same conclusion (Figure S1). Furthermore, the leave-one-out analysis suggested that the observed association remained non significantly altered after removing any single variant (Figure S2).

**Table 3:** The MR analysis about the association of HUA with the risk of DN

Exposure	MR method	SNPs	B	SE	OR(95%CI)	*P*
HUA	IVW	190	0.32	0.13	1.37 (1.07–1.76)	0.01
	MR-Egger	190	0.1	0.28	1.11 (0.64–1.91)	0.72
	Weighted median	190	0.37	0.16	1.45 (1.05–2.0)	0.02
	Weighted mode	190	0.65	0.25	1.91 (1.18–3.09)	< 0.01
	Simple mode	190	0.77	0.38	2.17 (1.02–4.59)	0.04

*OR* Odds Ratio, *CI* Confidence Interval, *SNP* single nucleotide polymorphisms, *IVW* inverse variance weighting, *HUA* *hyperuricemia*

## Discussion

In this study, we conducted the NHANES study and MR analysis to investigate the relationship between SUA and DN. This represents the first MR analysis conducted on this topic, and we utilized a large NHANES sample size. Our research results indicate that hyperuricemia is a contributing factor in the development of diabetic nephropathy in patients with diabetes.

UA is a byproduct of purine metabolism in the human body. Approximately two-thirds of UA is excreted through the kidneys, while the remaining one-third is excreted through the gastrointestinal tract. Its synthesis and excretion are typically balanced under physiological conditions. Any increase or decrease in UA excretion can result in elevated SUA levels. Normally, SUA levels range between 3.0 and 6.0 mg/dL [19]. Changes in UA production or decreased excretion can lead to hyperuricemia syndrome, commonly associated with conditions such as gout, tumor lysis, and urate crystal formation. These conditions can in turn lead to complications such as kidney stones, chronic kidney disease (CKD), gout nephropathy, secondary hypertension, and urinary tract infections [20].

The pathophysiological mechanism underlying the relationship between hyperuricemia and chronic complications is still unclear. One potential mechanism is that an increase in SUA levels leads to elevated reactive oxygen species (ROS) and promotes the expression of inflammatory cytokines, such as IL-1 β, IL-6, and TNF- α, which may cause vascular inflammation [21]. Additionally, research has found that SUA can induce endothelial dysfunction by inhibiting the migration and proliferation of endothelial cells, reducing the bioavailability of nitric oxide, and affecting the secretion of endothelial cells. This can activate the renin-angiotensin system and induce pro-inflammatory cytokines, leading to intraglomerular hypertension, vascular dysfunction, and inflammation, which in turn can contribute to cardiovascular and renal complications [9, 22]. Currently, studies have demonstrated an association between SUA and kidney disease, indicating a close relationship between hyperuricemia and kidney disease. Long-term hyperuricemia may lead to renal function damage and deterioration, and it is closely related to type 2 diabetic nephropathy (T2DN) [23].

Zoppini et al. [24] investigated the incidence rate of new CKD in 1449 T2D patients. During a 5-year follow-up, the cumulative incidence rate of CKD in patients with hyperuricemia was 29.5%, while in patients without hyperuricemia, it was 11.4% (*p* = 0.001). In univariate logistic regression analysis, hyperuricemia was associated with a 2.5-fold increase in CKD risk (OR 2.55 [95% CI 1.71–3.85], *p* = 0.001) [7]. After adjusting for age, gender, BMI, smoking, duration of T2DM, hypertension, insulin therapy, HbA1c, eGFR, and proteinuria, hyperuricemia remained associated with an increased risk of CKD (adjusted OR 2.10 [1.16–3.76], *p* = 0.01). In a longitudinal study in Italy, it was found that SUA is a powerful independent predictor of eGFR development in a large cohort of T2DM patients in real life. During a 4-year follow-up period, the highest gender-specific quintile of SUA levels led to a greater risk of eGFR compared to the lowest quintile of SUA (OR, 2.6) and was associated with proteinuria (OR, 1.5) [25].

A cross-sectional study of 343 Japanese men with type 2 diabetes found a positive correlation between SUA concentration and proteinuria [26]. Another cross-sectional study in China also indicated a positive correlation among SUA levels, proteinuria, and creatinine levels, but a negative correlation with glomerular filtration rate [27]. In a 3-year randomized controlled trial of losartan on patients with T2DM and chronic renal insufficiency stage 3–4 (RENAAL), it was observed that participants who experienced a decrease in serum uric acid levels after using losartan in the first 6 months had a lower risk of end-stage renal disease or sCr doubling compared to those who did not reduce their serum uric acid levels [28].

Contrary to this, some studies have indicated that there is no statistically significant causal relationship between hyperuricemia and DN. In one study, 393 patients with DN confirmed by biopsy were included. During the one-year follow-up, no significant correlation was found between the high SUA levels and the renal outcomes of Chinese diabetes patients and DN patients with end-stage renal disease [29]. Furthermore, the role of SUA lowering therapy in delaying DN progression and improving ESRD prognosis has not been confirmed. A recent randomized controlled trial in Australia and New Zealand showed that treatment with allopurinol to reduce SUA did not significantly reduce the decrease in eGFR compared to placebo, in multi-ethnic patients with high progression risk and stage 3–4 CKD [30]. Some studies have failed to demonstrate the statistical benefits of allopurinol on renal outcomes, indicating that even if SUA is reduced to lower levels, renal progression in T2DM patients does not improve as expected. This result may be explained by the predictive effect of SUA on renal function loss, which may not be direct and can be attributed to the causal correlation between SUA and other features related to DN, such as metabolic syndrome and insulin resistance [31].

Ahola et al. [32] found a significant correlation between elevated levels of SUA and worsening DN status in 2720 patients with type 1 diabetes. However, in an MR analysis using 25 known genetic predictors of SUA levels, no evidence of a causal relationship between SUA levels and DN progression was found. These findings suggest that the metabolic phenotype of SUA expression, such as overactive xanthine oxidase, rather than SUA itself, may be the reason for the increased risk of DN progression associated with elevated SUA levels.

High levels of SUA may exacerbate the development of DN. However, there are numerous factors that influence the occurrence and progression of DN, including various confounding factors like the duration of diabetes and blood sugar control. Unstable blood sugar control can lead to more serious vascular damage in diabetic patients, thereby worsening DN. The duration of diabetes also impacts the occurrence and development of kidney disease. Therefore, comparing the impact of uric acid on patients with DN will be greatly affected by these factors.

To minimize bias, we utilized various regression models to control for potential confounding factors. Across all models, we consistently observed that patients with hyperuricemia have a higher risk of developing diabetes nephropathy compared to those without hyperuricemia. Instrumental variables used for MR analysis are randomly allocated to the entire population during meiosis and conception, thereby reducing susceptibility to residual confounding and reverse causal bias. This method is considered a supplementary approach to RCT, as it uses genetic variables to assess the causal impact of exposure on specific outcomes.[18, 33].

## Advantages and limitations

One of the main advantages of our research is the use of a nationally representative large cohort to examine the relationship between SUA levels and the prevalence of DN. Additionally, we utilized two-sample Mendelian randomization methods to test for a causal relationship. However, our study has several limitations. First, we utilized the NHANES 2007–2016 database, which made it difficult to include all potential covariates, some of which may be outdated. Second, our study was cross-sectional, only measuring SUA once and not dynamically monitoring changes in uric acid or its impact on the progression of DN. Furthermore, for Mendelian randomization studies, all participants were from the European population, so caution should be exercised in applying the results to other races. Additionally, other confounding factors or environmental influences may impact the MR analysis. Finally, our study only investigated the causal relationship between SUA and DN, and further research is needed to explore the reverse causal relationship. Lastly, there is no distinction made between type 1 diabetes and type 2 diabetes in the diabetic patients included in this study.

## Conclusion

Through cross-sectional and MR analysis, we identified a strong causal relationship between hyperuricemia and diabetic nephropathy. We aim for our research to offer valuable evidence for the treatment of diabetes patients with hyperuricemia. Future prospective studies are needed to confirm the protective effect of uric acid-lowering therapy in diabetes patients, particularly those with diabetic nephropathy.

## Supplementary Information

Below is the link to the electronic supplementary material.

Supplementary file1 (DOCX 118 KB)

## Data Availability

The datasets used and/or analysed during the current study available from the corresponding author on reasonable request.
