# Supplementary material for: Association between hyperuricemia and diabetic nephropathy: insights from the national health and nutrition examination survey 2007–2016 and mendelian randomization analysis
Source: Int Urol Nephrol. 2024 May 29;56(10):3351–9. doi: 10.1007/s11255-024-04094-6 (PMC11405474; doi:10.1007/s11255-024-04094-6)

Table S1. Characteristics of genetic variants used to estimate the effect of SUA on DN.

| SNP | A1 | A2 | beta | eaf | se | pval |
| --- | --- | --- | --- | --- | --- | --- |
| rs10082474 | G | A | -0.0131 | 0.517038 | 0.0018 | 1.06E-13 |
| rs10196697 | A | G | -0.0105 | 0.502859 | 0.0019 | 2.71E-08 |
| rs10211562 | T | G | -0.0134 | 0.447795 | 0.0018 | 4.31E-14 |
| rs10279504 | A | C | -0.0122 | 0.277629 | 0.002 | 9.34E-10 |
| rs10405423 | A | C | 0.0216 | 0.721138 | 0.002 | 2.13E-26 |
| rs10429294 | T | C | 0.0205 | 0.577397 | 0.0018 | 4.10E-30 |
| rs1047891 | A | C | -0.0271 | 0.270739 | 0.002 | 1.54E-41 |
| rs10771025 | G | C | -0.0118 | 0.740902 | 0.002 | 7.17E-09 |
| rs10859925 | A | G | 0.0159 | 0.173649 | 0.0023 | 1.21E-11 |
| rs10886117 | A | G | 0.021 | 0.228111 | 0.0022 | 5.02E-22 |
| rs10901057 | G | C | 0.0172 | 0.846545 | 0.0027 | 2.36E-10 |
| rs10922199 | A | G | 0.0106 | 0.37727 | 0.0019 | 1.84E-08 |
| rs11056306 | A | G | -0.0108 | 0.429477 | 0.0018 | 1.17E-09 |
| rs11056396 | C | T | -0.0192 | 0.102439 | 0.0029 | 3.84E-11 |
| rs11065983 | A | C | -0.0313 | 0.173858 | 0.0034 | 3.29E-20 |
| rs11109717 | C | T | -0.0137 | 0.256941 | 0.002 | 2.18E-11 |
| rs11128603 | G | A | -0.0203 | 0.0964935 | 0.003 | 9.38E-12 |
| rs11163481 | T | G | 0.0125 | 0.643321 | 0.0018 | 1.24E-11 |
| rs11164916 | C | G | 0.0188 | 0.606666 | 0.0018 | 1.79E-25 |
| rs11202328 | T | C | -0.0254 | 0.273079 | 0.0023 | 2.11E-27 |
| rs112214565 | A | G | 0.0578 | 0.017376 | 0.0083 | 4.34E-12 |
| rs11243143 | A | G | -0.0353 | 0.63507 | 0.0021 | 5.13E-63 |
| rs11264341 | T | C | -0.0352 | 0.516603 | 0.0018 | 1.57E-82 |
| rs114158982 | G | C | 0.0584 | 0.017469 | 0.0077 | 4.94E-14 |
| rs114165349 | C | G | 0.0805 | 0.023533 | 0.0067 | 8.01E-33 |
| rs11564722 | T | C | -0.0204 | 0.297748 | 0.002 | 1.45E-24 |
| rs1169288 | C | A | -0.0202 | 0.362519 | 0.0019 | 1.83E-27 |
| rs1171614 | C | T | 0.0563 | 0.76883 | 0.0024 | 1.73E-120 |
| rs11827106 | A | G | -0.0124 | 0.218584 | 0.0022 | 3.33E-08 |
| rs11871152 | G | A | 0.0105 | 0.44171 | 0.0018 | 4.67E-09 |
| rs11940694 | G | A | 0.0106 | 0.555862 | 0.0018 | 3.68E-09 |
| rs12070208 | C | T | -0.0211 | 0.0808117 | 0.0038 | 3.16E-08 |
| rs12096443 | T | C | -0.0174 | 0.346507 | 0.0019 | 1.54E-19 |
| rs12105304 | G | A | 0.0206 | 0.236609 | 0.0021 | 7.91E-22 |
| rs12158162 | G | A | -0.0212 | 0.0595896 | 0.0037 | 1.20E-08 |
| rs12277177 | G | A | 0.0157 | 0.17535 | 0.0027 | 4.12E-09 |
| rs12467636 | G | A | 0.013 | 0.755523 | 0.0021 | 3.59E-10 |
| rs12510175 | G | C | -0.0117 | 0.424792 | 0.0019 | 3.45E-10 |
| rs12543287 | C | G | -0.0115 | 0.365126 | 0.0018 | 3.85E-10 |
| rs12575710 | A | G | 0.0264 | 0.208351 | 0.0024 | 4.16E-29 |
| rs1260326 | C | T | -0.0445 | 0.561756 | 0.0018 | 1.88E-136 |
| rs12666237 | T | C | 0.0202 | 0.10066 | 0.003 | 3.04E-11 |
| rs12708477 | C | A | -0.0124 | 0.802148 | 0.0022 | 1.98E-08 |
| rs12767261 | A | G | 0.0166 | 0.106677 | 0.0029 | 6.67E-09 |
| rs12790943 | T | C | -0.014 | 0.306542 | 0.0021 | 8.71E-12 |
| rs12806743 | T | G | -0.0138 | 0.219438 | 0.0021 | 9.56E-11 |
| rs12887732 | A | C | 0.0114 | 0.724795 | 0.002 | 8.45E-09 |
| rs12891886 | C | G | 0.013 | 0.240285 | 0.0021 | 3.23E-10 |
| rs12911430 | T | A | 0.0123 | 0.316795 | 0.002 | 1.82E-09 |
| rs12937692 | A | G | 0.0131 | 0.284543 | 0.002 | 1.49E-10 |
| rs12973608 | C | A | -0.014 | 0.67689 | 0.0019 | 1.99E-13 |
| rs12979148 | C | T | 0.0149 | 0.143599 | 0.0025 | 3.48E-09 |
| rs12987661 | C | T | -0.0302 | 0.100501 | 0.0029 | 4.22E-25 |
| rs12992672 | A | G | 0.0156 | 0.84873 | 0.0024 | 2.10E-10 |
| rs13107325 | T | C | -0.031 | 0.074844 | 0.0039 | 9.97E-16 |
| rs1317983 | C | T | 0.0304 | 0.744566 | 0.002 | 2.65E-50 |
| rs13230509 | C | G | 0.0314 | 0.58922 | 0.0019 | 2.61E-58 |
| rs13247874 | T | C | -0.0345 | 0.171856 | 0.0023 | 1.99E-49 |
| rs13411042 | A | C | 0.0145 | 0.444883 | 0.0018 | 4.33E-16 |
| rs13418518 | T | A | 0.0366 | 0.032189 | 0.0058 | 2.22E-10 |
| rs140753856 | G | A | -0.0147 | 0.150225 | 0.0025 | 6.14E-09 |
| rs140885868 | G | A | -0.0893 | 0.013354 | 0.0097 | 2.40E-20 |
| rs145157727 | A | G | -0.0518 | 0.0114684 | 0.0082 | 2.98E-10 |
| rs148179165 | A | C | 0.0164 | 0.346904 | 0.0019 | 3.20E-18 |
| rs148688688 | A | G | 0.0426 | 0.0214268 | 0.0065 | 4.42E-11 |
| rs150147865 | T | A | -0.1163 | 0.0088936 | 0.0116 | 8.70E-24 |
| rs1511299 | C | T | -0.0211 | 0.266849 | 0.002 | 4.15E-26 |
| rs157512 | C | T | -0.0202 | 0.23748 | 0.0025 | 7.68E-16 |
| rs1622987 | A | G | 0.011 | 0.275551 | 0.002 | 4.54E-08 |
| rs16942751 | A | C | 0.0176 | 0.146172 | 0.0027 | 3.21E-11 |
| rs17013743 | G | A | -0.0298 | 0.0793353 | 0.0034 | 8.66E-19 |
| rs17024258 | T | C | 0.036 | 0.02586 | 0.0064 | 2.12E-08 |
| rs17050272 | A | G | 0.0218 | 0.429899 | 0.0018 | 1.59E-34 |
| rs1719985 | T | C | -0.0118 | 0.406236 | 0.0018 | 1.60E-10 |
| rs17592117 | C | T | 0.0426 | 0.0916586 | 0.0031 | 1.80E-43 |
| rs17624477 | C | T | 0.0252 | 0.0551457 | 0.0038 | 5.10E-11 |
| rs17632159 | C | G | -0.0299 | 0.299966 | 0.0019 | 3.10E-54 |
| rs17786744 | G | A | 0.0223 | 0.365981 | 0.0018 | 1.16E-33 |
| rs181673 | C | A | 0.0173 | 0.590031 | 0.0018 | 1.56E-21 |
| rs1851285 | G | C | 0.013 | 0.551001 | 0.0018 | 1.34E-12 |
| rs1869581 | C | G | -0.0109 | 0.444542 | 0.0018 | 2.00E-09 |
| rs187355703 | G | C | 0.0563 | 0.0191647 | 0.0064 | 1.32E-18 |
| rs1925258 | A | T | -0.0124 | 0.665384 | 0.0019 | 5.43E-11 |
| rs1955949 | C | T | -0.0137 | 0.75259 | 0.002 | 2.39E-11 |
| rs2039424 | A | G | 0.0111 | 0.638438 | 0.0018 | 1.65E-09 |
| rs2057291 | G | A | -0.0146 | 0.673553 | 0.0019 | 5.75E-15 |
| rs2060824 | C | T | 0.0135 | 0.480515 | 0.0018 | 3.28E-14 |
| rs2075252 | C | T | -0.0261 | 0.672548 | 0.002 | 1.69E-40 |
| rs2108093 | G | A | 0.0151 | 0.829163 | 0.0023 | 1.19E-10 |
| rs2195525 | T | C | -0.014 | 0.600575 | 0.0019 | 6.72E-14 |
| rs219777 | A | G | -0.0227 | 0.24762 | 0.0023 | 4.19E-22 |
| rs2210315 | T | C | 0.0181 | 0.17134 | 0.0027 | 2.27E-11 |
| rs2219647 | A | G | 0.0163 | 0.255685 | 0.002 | 1.25E-15 |
| rs2229357 | A | G | -0.0449 | 0.199117 | 0.0022 | 1.12E-90 |
| rs2231145 | C | T | -0.0787 | 0.0426661 | 0.0046 | 3.35E-64 |
| rs2240193 | A | C | -0.0258 | 0.356532 | 0.0037 | 2.09E-12 |
| rs2240390 | C | T | -0.0132 | 0.325505 | 0.0019 | 7.32E-12 |
| rs2252862 | G | C | -0.0147 | 0.470181 | 0.0019 | 6.01E-15 |
| rs2287084 | T | G | 0.0124 | 0.228085 | 0.0021 | 6.36E-09 |
| rs2362515 | A | G | -0.0121 | 0.314821 | 0.0019 | 2.30E-10 |
| rs2414064 | A | G | -0.0144 | 0.21361 | 0.0025 | 7.10E-09 |
| rs2436958 | C | T | -0.0188 | 0.828966 | 0.0024 | 1.44E-15 |
| rs2437817 | A | C | -0.0146 | 0.309596 | 0.0019 | 2.60E-14 |
| rs2480714 | T | G | 0.0126 | 0.765931 | 0.0022 | 5.87E-09 |
| rs2493121 | A | T | 0.0121 | 0.711628 | 0.002 | 1.05E-09 |
| rs2540034 | T | C | 0.0109 | 0.502628 | 0.0018 | 3.42E-09 |
| rs2581824 | A | C | 0.03 | 0.446643 | 0.0018 | 4.94E-64 |
| rs2636590 | A | G | 0.011 | 0.631534 | 0.0018 | 2.29E-09 |
| rs2644128 | G | C | 0.0136 | 0.471319 | 0.0018 | 7.44E-14 |
| rs2668423 | G | T | -0.0115 | 0.734607 | 0.002 | 1.09E-08 |
| rs2695580 | A | G | 0.0107 | 0.370913 | 0.0019 | 1.33E-08 |
| rs2714345 | A | G | -0.0122 | 0.228012 | 0.0021 | 1.39E-08 |
| rs272889 | G | A | 0.013 | 0.544845 | 0.0018 | 1.85E-12 |
| rs2749005 | G | T | 0.0116 | 0.708888 | 0.002 | 4.67E-09 |
| rs2762353 | G | A | 0.0668 | 0.642554 | 0.0019 | 1.00E-200 |
| rs2788144 | G | A | 0.0395 | 0.03673 | 0.0054 | 3.38E-13 |
| rs2808500 | G | A | -0.0104 | 0.433211 | 0.0018 | 5.89E-09 |
| rs2823139 | A | G | 0.0152 | 0.314419 | 0.0019 | 2.41E-15 |
| rs2834317 | A | G | 0.0192 | 0.127508 | 0.0027 | 4.91E-13 |
| rs28419182 | A | G | 0.018 | 0.694159 | 0.002 | 2.87E-19 |
| rs28517717 | T | C | -0.0218 | 0.338371 | 0.0019 | 9.78E-30 |
| rs2926590 | A | C | 0.0193 | 0.182865 | 0.0023 | 5.10E-17 |
| rs2941484 | T | C | 0.0318 | 0.443189 | 0.0018 | 1.30E-71 |
| rs2943645 | T | C | 0.0134 | 0.719598 | 0.002 | 2.96E-11 |
| rs2973444 | C | T | -0.0245 | 0.0636752 | 0.0036 | 1.07E-11 |
| rs3116068 | T | C | 0.0184 | 0.212027 | 0.0022 | 1.67E-17 |
| rs3212198 | T | C | -0.0172 | 0.503289 | 0.0018 | 4.80E-21 |
| rs33938520 | T | C | -0.0135 | 0.25276 | 0.0023 | 9.79E-09 |
| rs34381009 | G | A | -0.0387 | 0.169001 | 0.0023 | 2.10E-61 |
| rs34811474 | A | G | -0.014 | 0.23213 | 0.0024 | 6.04E-09 |
| rs34868798 | C | T | -0.0141 | 0.22033 | 0.0024 | 7.87E-09 |
| rs35523212 | G | A | -0.0448 | 0.518307 | 0.0018 | 7.64E-130 |
| rs35994853 | T | C | 0.0151 | 0.370769 | 0.0019 | 1.44E-15 |
| rs3824359 | C | T | 0.0142 | 0.175528 | 0.0023 | 1.53E-09 |
| rs3845534 | A | G | 0.0151 | 0.581636 | 0.0018 | 3.57E-16 |
| rs429358 | C | T | -0.0182 | 0.141145 | 0.0025 | 6.05E-13 |
| rs4468717 | T | C | -0.0214 | 0.077674 | 0.0038 | 1.74E-08 |
| rs4476815 | C | G | 0.0366 | 0.0261919 | 0.0058 | 2.29E-10 |
| rs45487598 | A | G | 0.0184 | 0.11689 | 0.0032 | 7.70E-09 |
| rs45499402 | C | G | 0.1462 | 0.163764 | 0.0024 | 1.00E-200 |
| rs455213 | C | T | 0.0165 | 0.402199 | 0.0018 | 1.44E-19 |
| rs4575545 | A | G | -0.0267 | 0.298394 | 0.0019 | 2.23E-43 |
| rs4777466 | T | C | -0.0149 | 0.355818 | 0.0019 | 1.84E-14 |
| rs4783505 | T | G | 0.0139 | 0.461732 | 0.0019 | 6.18E-13 |
| rs4789278 | A | G | -0.0179 | 0.264277 | 0.0022 | 1.35E-15 |
| rs4886755 | G | A | 0.0285 | 0.506423 | 0.0018 | 2.84E-57 |
| rs4897160 | A | G | 0.0145 | 0.477568 | 0.0018 | 1.79E-16 |
| rs4966019 | T | C | -0.0323 | 0.599258 | 0.0018 | 1.10E-70 |
| rs496708 | A | C | -0.0133 | 0.447339 | 0.0018 | 7.26E-13 |
| rs505870 | A | C | -0.013 | 0.47511 | 0.002 | 1.78E-10 |
| rs538737 | G | C | 0.0786 | 0.433648 | 0.002 | 1.00E-200 |
| rs57577420 | G | A | 0.0187 | 0.110959 | 0.0028 | 4.62E-11 |
| rs589852 | T | C | -0.0288 | 0.596218 | 0.0018 | 3.84E-58 |
| rs60767324 | C | T | -0.0189 | 0.0763012 | 0.0033 | 1.20E-08 |
| rs6129786 | A | C | -0.013 | 0.358439 | 0.0021 | 3.40E-10 |
| rs61903695 | G | A | 0.012 | 0.216906 | 0.0021 | 2.13E-08 |
| rs61941404 | T | C | -0.028 | 0.071351 | 0.0037 | 2.41E-14 |
| rs62106258 | C | T | -0.0342 | 0.048343 | 0.0047 | 5.11E-13 |
| rs62262727 | A | G | 0.0358 | 0.069758 | 0.004 | 3.53E-19 |
| rs62294340 | A | G | -0.017 | 0.374617 | 0.0018 | 9.99E-21 |
| rs62580766 | T | C | -0.0144 | 0.18197 | 0.0026 | 4.75E-08 |
| rs6429747 | T | C | -0.0215 | 0.31423 | 0.0022 | 9.60E-23 |
| rs6464165 | C | T | 0.0267 | 0.28282 | 0.0023 | 3.79E-32 |
| rs6492910 | A | G | -0.0133 | 0.641321 | 0.002 | 2.02E-11 |
| rs6674490 | A | C | -0.0123 | 0.393648 | 0.0018 | 9.68E-12 |
| rs6715785 | A | G | -0.0116 | 0.415762 | 0.0021 | 3.98E-08 |
| rs676015 | C | T | -0.015 | 0.664388 | 0.0019 | 1.88E-15 |
| rs680071 | C | T | 0.0158 | 0.869594 | 0.0026 | 1.50E-09 |
| rs6804946 | C | T | -0.0216 | 0.20022 | 0.0025 | 1.77E-17 |
| rs6825697 | C | G | 0.0365 | 0.917691 | 0.0038 | 1.12E-21 |
| rs6860575 | T | C | -0.0114 | 0.472925 | 0.0018 | 1.88E-10 |
| rs686364 | G | A | 0.0167 | 0.286199 | 0.002 | 3.10E-17 |
| rs6939175 | A | G | -0.012 | 0.287096 | 0.002 | 3.60E-09 |
| rs7039 | G | C | 0.0147 | 0.550652 | 0.0018 | 2.49E-16 |
| rs703978 | G | C | 0.0117 | 0.615663 | 0.0018 | 1.11E-10 |
| rs7093087 | A | G | 0.0172 | 0.163333 | 0.0024 | 4.35E-13 |
| rs7122026 | A | C | 0.0121 | 0.364756 | 0.002 | 1.39E-09 |
| rs7188156 | G | T | 0.0276 | 0.168983 | 0.0024 | 2.52E-31 |
| rs7200986 | A | G | -0.0135 | 0.38712 | 0.0018 | 3.00E-13 |
| rs7224610 | A | C | -0.0265 | 0.662467 | 0.0019 | 6.51E-44 |
| rs7230932 | A | G | -0.0101 | 0.359474 | 0.0018 | 4.09E-08 |
| rs7247977 | C | T | 0.0176 | 0.469395 | 0.0018 | 2.47E-22 |
| rs72681698 | C | T | -0.0717 | 0.011039 | 0.0097 | 1.63E-13 |
| rs72818964 | A | G | 0.0155 | 0.17609 | 0.0027 | 5.59E-09 |
| rs72951456 | T | C | 0.0264 | 0.048935 | 0.0048 | 3.17E-08 |
| rs7302925 | G | A | -0.0184 | 0.827202 | 0.0023 | 2.30E-15 |
| rs7310615 | G | C | -0.0289 | 0.5182 | 0.002 | 3.63E-45 |
| rs73169739 | C | T | 0.0242 | 0.0622486 | 0.0036 | 2.90E-11 |
| rs7331398 | C | T | -0.0148 | 0.503392 | 0.0019 | 1.37E-14 |
| rs738409 | G | C | -0.0199 | 0.281751 | 0.002 | 7.17E-23 |
| rs7402939 | C | T | 0.012 | 0.587977 | 0.0018 | 3.52E-11 |
| rs74397112 | T | C | 0.0227 | 0.103764 | 0.0029 | 5.19E-15 |
| rs74606487 | G | A | -0.0148 | 0.134383 | 0.0027 | 3.95E-08 |
| rs753009 | T | C | 0.0142 | 0.239402 | 0.0021 | 3.63E-11 |
| rs754600 | A | G | 0.0152 | 0.295842 | 0.0021 | 1.31E-12 |
| rs75523587 | A | T | 0.0174 | 0.206794 | 0.0022 | 1.94E-15 |
| rs7586329 | C | T | 0.0641 | 0.992254 | 0.0116 | 3.25E-08 |
| rs7642977 | T | C | 0.0106 | 0.416168 | 0.0019 | 3.94E-08 |
| rs7651369 | G | A | -0.011 | 0.477149 | 0.0019 | 1.31E-08 |
| rs7677783 | T | C | -0.0114 | 0.610034 | 0.0018 | 3.34E-10 |
| rs76895963 | G | T | -0.0557 | 0.021069 | 0.0078 | 9.75E-13 |
| rs7696556 | C | A | -0.0168 | 0.274427 | 0.0023 | 2.48E-13 |
| rs77008184 | G | A | 0.0188 | 0.100256 | 0.0029 | 1.70E-10 |
| rs7766720 | C | T | 0.021 | 0.106151 | 0.0029 | 6.35E-13 |
| rs7773175 | G | C | -0.0216 | 0.271964 | 0.002 | 6.52E-28 |
| rs77924615 | A | G | -0.0151 | 0.202983 | 0.0022 | 9.05E-12 |
| rs784257 | C | T | 0.0149 | 0.81295 | 0.0026 | 1.34E-08 |
| rs78671965 | T | A | 0.0271 | 0.055477 | 0.0045 | 1.73E-09 |
| rs7952306 | T | G | 0.025 | 0.780974 | 0.0024 | 6.14E-26 |
| rs8039645 | A | C | 0.0127 | 0.206227 | 0.0022 | 1.01E-08 |
| rs807624 | T | G | -0.0168 | 0.477412 | 0.0019 | 8.23E-19 |
| rs831036 | G | C | 0.0146 | 0.396339 | 0.0018 | 7.48E-16 |
| rs833805 | G | A | 0.0226 | 0.882604 | 0.0028 | 1.47E-15 |
| rs836968 | T | C | -0.014 | 0.34602 | 0.0019 | 3.59E-13 |
| rs856560 | C | T | 0.0133 | 0.704475 | 0.002 | 2.74E-11 |
| rs926979 | C | T | 0.0137 | 0.716224 | 0.002 | 3.35E-12 |
| rs9307594 | G | A | 0.0105 | 0.575262 | 0.0019 | 2.92E-08 |
| rs9333592 | T | C | -0.0239 | 0.078094 | 0.0039 | 5.83E-10 |
| rs9534949 | G | C | -0.0133 | 0.708275 | 0.0019 | 6.51E-12 |
| rs963837 | C | T | -0.0245 | 0.42566 | 0.0018 | 4.72E-43 |
| rs9807214 | A | G | 0.0128 | 0.240309 | 0.0021 | 1.32E-09 |
| rs9843304 | T | C | 0.0103 | 0.530975 | 0.0018 | 5.07E-09 |
| rs9880232 | A | C | -0.0144 | 0.774593 | 0.0022 | 3.93E-11 |

Figure S1. Funnel plots to visualize the overall heterogeneity of MR estimates


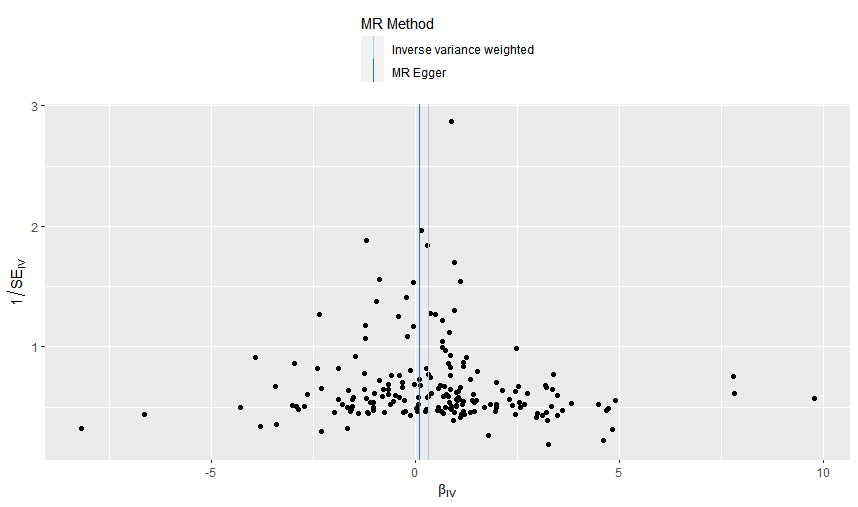


Figure S2. Leave-one-out plot from genetically predicted this bidirectional MR.


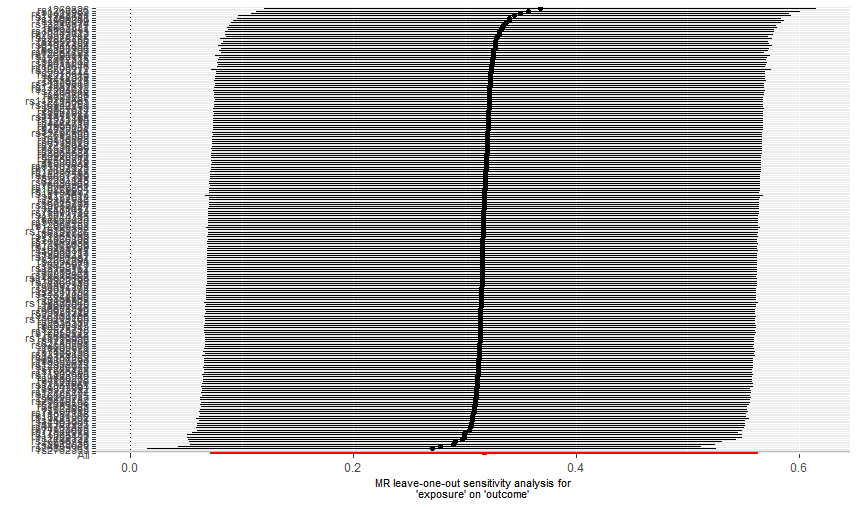

Supplement: Supplementary file 1 — Supplementary file1 (DOCX 118 KB) [file 11255_2024_4094_MOESM1_ESM.docx]
